# One palatal implant for skeletal anchorage – frequency and range of indications

**DOI:** 10.1186/s13005-015-0073-x

**Published:** 2015-04-21

**Authors:** Elena Krieger, Zeynep Yildizhan, Heinrich Wehrbein

**Affiliations:** Department of Orthodontics, University Medical Centre of the Johannes Gutenberg University Mainz, Augustusplatz 2, 55131 Mainz, Germany

**Keywords:** Palatal implant, Ortho system, Orthodontic treatment, Indication, Frequency, Skeletal anchorage

## Abstract

**Objective:**

Aim of this investigation was to analyze the frequency and range of indications of orthodontic treatments using one palatal implant for skeletal anchorage, in a time frame of four years.

**Material and methods:**

A sample was comprised by viewing retrospectively the patient collective of a specialized university clinic who started orthodontic treatment in the time frame 01/09-12/12. Inclusion criterion was the first application of a superstructure within the investigated period after successful insertion of a palatal implant (Ortho-System®, Straumann, Basel, Switzerland). Frequency and range of indications of the conducted skeletally anchored tooth movement were determined by analyzing the individual patient documentation such as medical records, radiographs and casts.

**Results:**

From a total of 1350 patients who started orthodontic treatment in this period met 56 (=4.2%) the inclusion criterion. In 85.7% of this sample was sagittal orthodontic tooth movement conducted, most frequently mesialization of ≥1 tooth (44.6%). Vertical tooth movement was in 57.1% of the sample performed, mostly extrusion of ≥1 tooth (34%). In 33.9% of the sample was ≥1 displaced tooth orthodontically relocated. One or two upper incisors were in 16.1% of the sample permanently replaced by the superstructure, all but one even after orthodontic treatment. In 66.1% of all cases were multi-functional anchorage challenges performed.

**Conclusion:**

4.2 % of all treated patients within the investigated period required orthodontic treatment with skeletal anchorage (palatal implant), mainly for performing sagittal tooth movement (mesialization). The palatal implant was primarily used for multi-functional anchorage purposes, including skeletally anchored treatment in the mandible.

## Introduction

**Figure 1:**
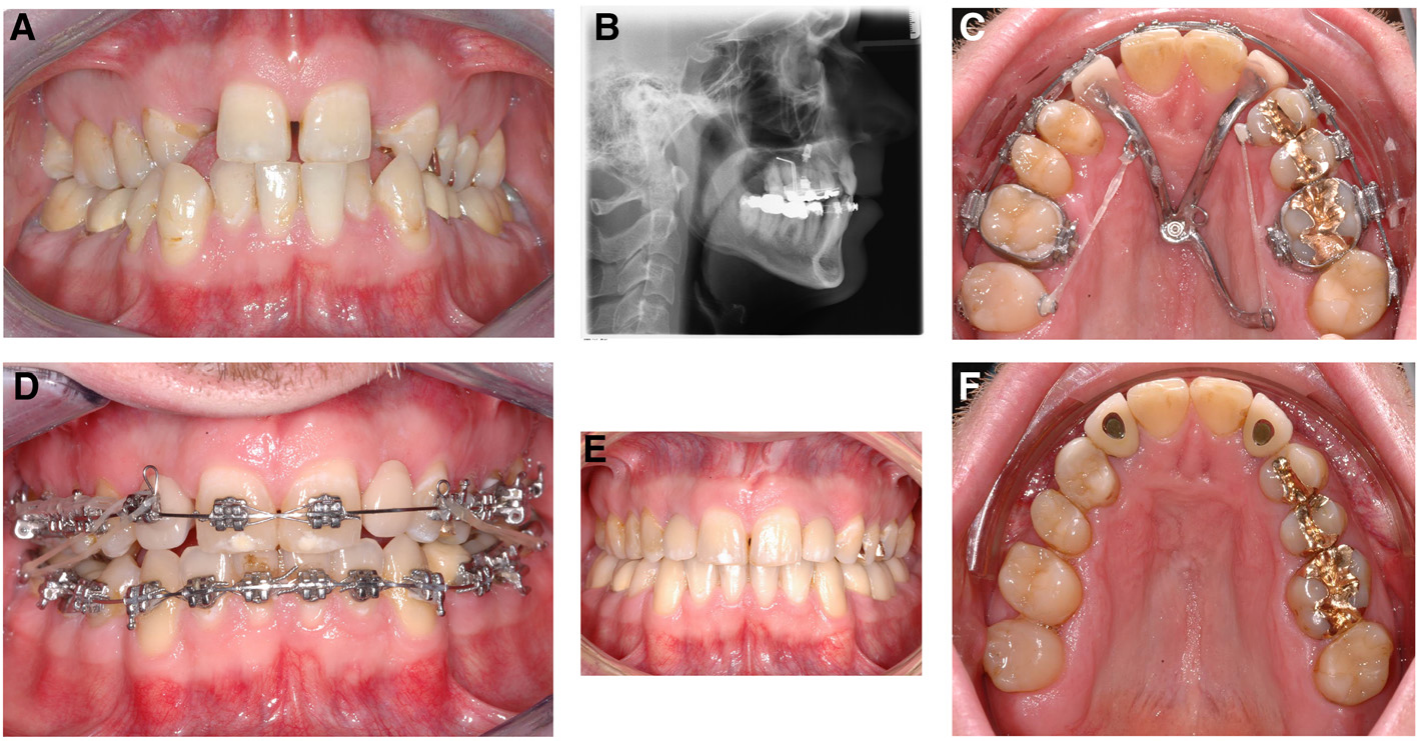
Patient example of a 28y old male; **A)** Diagnosis: previous orthodontic treatment as a child; missing both upper lateral incisors and canines; maxillary transversal deficit; anterior crowding in the mandible; anterior edge-to-edge bite; **B)** Lateral cephalogram with an inserted PI, after a previously performed surgically assisted rapid maxillary expansion (SRME). **C)** Multi-functional anchorage purposes: mesialization of the right upper lateral teeth, distalization of the left upper lateral teeth, replacement of missing upper lateral incisors. **D)** Applied Class II elastics for mesialization of the lower teeth for gap closure (after extraction of the first premolars to resolve the anterior crowding). **E** and **F)** Final situation after removal of the orthodontic appliances and insertion of dental implants in the region of the upper lateral incisors. The first upper premolars were shaped and build up with composite to fit as a replacement for the canines.

The development of moving teeth with skeletal anchorage created more opportunities for orthodontic treatment. So-called TADs (= Temporary Anchorage Devices) are temporarily inserted bone-borne mini-implants or mini-plates, used only for orthodontic treatment purposes and usually removed after finishing the orthodontic treatment. They can be divided into three groups: mini-screws (= MS; diameter reduces mini-implants, inserted in the alveolar bone or anterior palate), bone anchors (= BA; mini-plates, inserted in the maxillary or mandibular basal bone) and palatal implants (=PI; length reduced mini-implants, inserted in the anterior palatal, Figure 1B). Due to the failure rate of MS inserted in the alveolar bone, torque-resisting TADs (BA and PI) became more of interest; they showed a 1.92-fold lower clinical failure rate than MS [1]. In addition, aspects like the higher bone density of the palatal bone [2] compared to the alveolar bone aroused the anterior palate as an insertion site of interest, even as an insertion site for MS. Jung et al. demonstrated 2012 in their multicenter investigation a failure rate of 4.6 % (n = 239) when inserting a PI [3]. Rodriguez et al. performed 2014 a literature research of failure rates of TADs and found that survival rates of MS were location dependent, but those placed in the anterior palate showed higher success rates [4].

A MS (inserted in the anterior palate) or a PI provides a connection to receive a superstructure after successful insertion [5-8]. The procedure is similar to conventional dental implants, after taking an impression including an impression post and producing a cast within an implant transfer, a superstructure can be created, designed for the individual anchorage purposes (Figure 1C).

Without the need to depend or rely on using the residual dentition or extraoral appliances (e.g. headgear) as anchorage the opportunity to treat even patients with bone loss (previous periodontal diseases) or without compliance is given. Previously, various investigations reported about failure or survival rates [1,3,9], regarding individual techniques of insertion [10] or fabricate design [11]. About several indications for orthodontic treatment with skeletal anchorage has been reported [7,8,12], often described in case reports [13,14] and discussed in reviews [15,16] or experts opinions [17-21]. But nevertheless, it has not yet been clarified in how many cases the use of skeletal anchorage is actually necessary and when, which indications should to be placed.

Therefore we conducted this investigation to analyze the frequency of orthodontic treatment with skeletal anchorage (using one palatal implant), as well as the range of indications in a time frame of four years.

## Material and methods

At first a sample was comprised by viewing retrospectively all patients from a specialized university clinic who started orthodontic treatment in the period January 2009 to December 2012, regardless of age and gender.

Further selection was the successful insertion of a palatal implant (second generation, endosseous portion: length, 4.2 mm; diameter, 4.1 mm; Ortho-System®, Straumann, Basel, Switzerland) (Figure 1B) i.e. successful healing period, no failures or re-insertions. The main inclusion criterion for patient recruitment was the first application of a superstructure on the PI (conventional loading) in the defined time frame. All patients had to be healthy, patients presenting multiple agenesis (>2 agenesis per quadrant), or a cleft lip or palate, or any other syndromic orofacial malformations were excluded, because they received a special treatment protocol.

After recruitment of the sample we analyzed the frequency and range of indications by using patient documentation (medical records, radiographs, plaster models). We collected patients’ as well as appliance specific data. The frequency of complications i.e. reparation of the superstructure and loss of the bonding patches were collected.

The sample was divided into three subclasses regarding the individual functionality of the superstructure: 1) uni-functional, 2) bi-functional and 3) multi-functional treatment purposes. Uni-functionality was defined as one force vector loaded to the PI, bi-functional as two forces and multi-functionality > 2 vectors.

Indications were placed dividing the design of the superstructure/orthodontic tooth movement into the following parameters:

1. Sagittal tooth movement: a) mesialization of ≥ 1 tooth, b) distalization of ≥ 1 tooth, c) both (simultaneous mesialization and distalization of different teeth).
2. Vertical tooth movement: a) extrusion of ≥ 1 tooth, b) intrusion of ≥ 1 tooth, c) both (simultaneous extrusion and intrusion of different teeth), d) orthodontic treatment of displaced teeth.
3. Temporary replacement of missing (anterior) teeth.

No assessment of the treatment outcome was conducted; therefore no statement of the efficiency can be made. This investigation was an exclusively descriptive analysis of existing material. All patients gave their consent to use their patient documentation for internal analysis and assessment before any treatment was performed. After recruitment the sample, all data were anonymized for further investigation. The local ethical committee (State Chamber of Medicine in Rheinland-Pfalz, Germany) gave its approval for retrospective, anonymized studies on the 14th of January 2015.

### Statistical analysis

The collection and descriptive analysis of these data were carried out using SPSS software (Statistical Package for Social Science) for Windows, version 21.0 (SPSS Software Corp., Chicago, IL, USA). The evaluation was performed as a descriptive analysis of continuous variables by specifying the statistical parameters of mean, minimum, maximum, and standard deviation (SD), and on the basis of relative frequencies.

## Results

### Subjects

From a total of 1350 patients who started orthodontic treatment within the investigated period, 56 (n = 4.2%) met the inclusion criterion. The patients’ mean age was 19.5y (11-52y). 60.7% (n = 34) of the sample were females and 39.3% (n = 22) males. No implant was lost during the investigated period.

In 75% of the sample (n = 42) was one superstructure inserted, 23.2% (n = 13) received two and 1.8% (n = 1) three.

In 71.4% (n = 40) of the 56 subjects was the skeletal anchorage solely used in the maxilla, in 28.6% (n = 16) also for treatment functions in the mandible (i.e. mesialization of lateral teeth in the mandible to perform gap closure by applying Class II elastics, Figure 1D). In detail, most of patients were treated in two quadrants (first and second quadrant) (48.2%, n = 27), but secondly 21.4% (n = 12) were treated in all 4 quadrants (Tables 1 and 2).

**Table 1:** **Number of orthodontically treated quadrants per patient (n = 56), in absolute and relative frequencies**

	**Number of quadrants** ***(each subject, n = 56)***	
	**n**	**%**
One quadrant	13	23.2
Two quadrants	27	48.2
Three quadrants	4	7.1
Four quadrants	12	21.4

**Table 2:** **Orthodontic treatment per quadrant of each patient (n = 56), in absolute and relative frequencies**

**Quadrants**	**Treatment per quadrant** ***(n = 56)***	
	**n**	**%**
Upper right	6	10.7
Upper left	7	12.5
Upper right plus left	27	48.2
Upper right plus left and lower right	0	0
Upper right plus left and lower left	4	7.1
Upper right plus left and lower right plus left	12	21.4

### Complications

The superstructure had to be repaired in 5.4 % of all cases. Partial surfaces of the teeth were bonded to the superstructure when using indirect anchorage to move the teeth. Re-bonding after loss of the bonding patch had to be done in 23.2% (n = 13) of the cases. Evaluating in which indications the loss occurred and which provider conducted the treatment we found that nearly all incidents occurred when treating displaced upper canines, conducted by the same provider.

### Functionality

Regarding the individual treatment purposes of the superstructure, 7.1 % (n = 4) of the cases were uni-functional, i.e. only one force vector loaded to the PI, 26.8% (n = 15) bi-functional and 66.1% (n = 37) multi-functional (Figure 1C).

### Indications

#### Sagittal tooth movement

**Figure 2:**
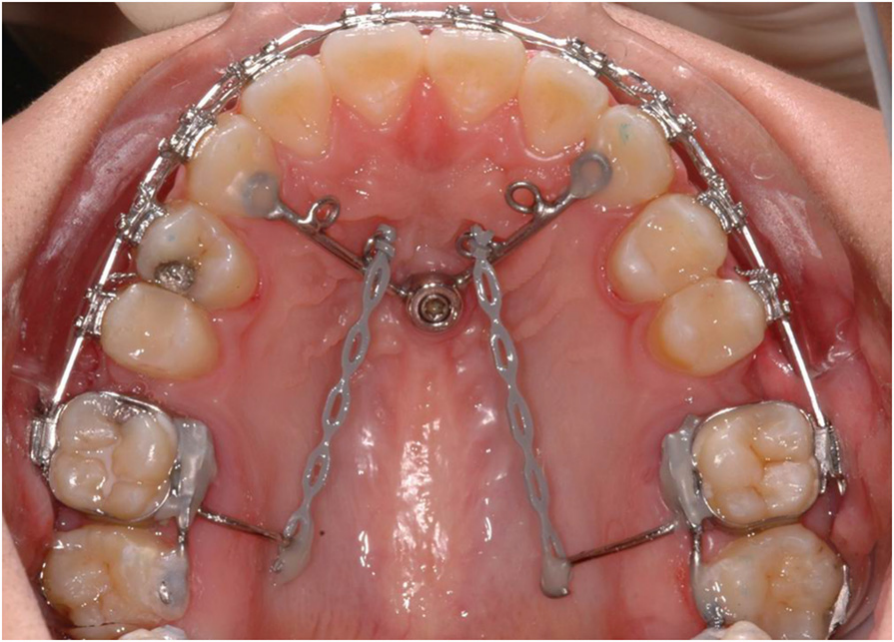
Patient example of a superstructure to mesialize all upper and lower second and third molars.

**Figure 3:**
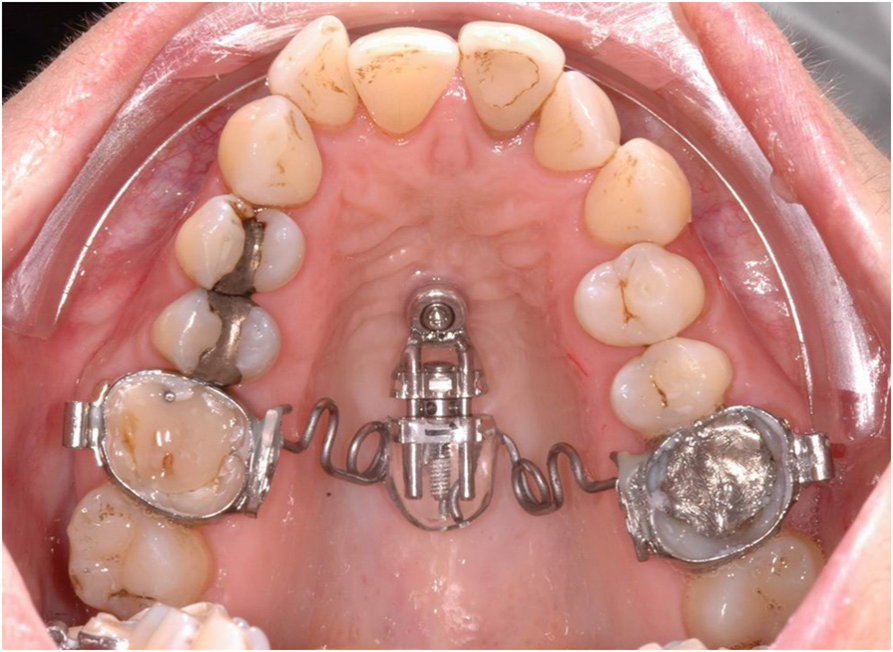
Patient example of a superstructure to distalize the upper lateral teeth (“skeletally supported Pendulum appliance”).

In 85.7% (n = 48) of the sample (n = 56) was sagittal tooth movement conducted (Table 3, Figure 1C). Mesialization of lateral teeth was in 28.6% (n = 16) also performed in the mandible (Figure 1D). Different types of appliances were used for mesialization (patient example, Figure 2) and distalization (patient example, Figure 3).

**Table 3:** **Sagittal tooth movement: patients in whom orthodontic sagittal tooth movement was performed (n = 48), subdivided into mesialization, distalization and simultaneous mesialization and distalization of different teeth; in total and in relation to the whole sample; in absolute and relative frequencies**

	**Patients with sagittal movement** ***(n = 48)***		**All patients** ***(n = 56)***	
	**n**	**%**	**n**	**%**
Mesialization ≥1 tooth	25	52.1	25	44.6
Distalization ≥1 tooth	13	27.1	13	23.2
Mesialization plus distalization	10	20.8	10	17.9

#### Vertical tooth movement/treatment of displaced teeth

Vertical tooth movement was conducted in 57.1 % (n = 32) (Table 4), a combination of vertical and sagittal movement in 44.6 % (n = 25).

**Table 4:** **Vertical tooth movement: patients who underwent vertical tooth movement (n = 32), subdivided and in relation to the whole sample (n = 56), in absolute and relative frequencies**

	**Patients with vertical movement** ***(n = 32)***		**All patients** ***(n = 56)***	
	**n**	**%**	**n**	**%**
Extrusion ≥1 tooth	19	59.4	19	34
Intrusion ≥1 tooth	8	25	8	14.3
Extrusion plus intrusion	5	15.6	5	8.9

In 33.9% of the sample (n = 19) was ≥1 tooth displaced. On average 1.6 teeth were displaced (Min.1, Max. 4), most frequently one or both upper canines, and orthodontically relocated using skeletal anchorage.

#### Temporary replacement of missing teeth

In 16.1 % (n = 9) of all cases were one (n = 6) or two upper incisors (n = 3) temporarily replaced during orthodontic treatment (Figure 1C), apart from one patient even after active orthodontic treatment as a permanent replacement.

## Discussion

Skeletal anchorage for orthodontic treatment purposes has been part of many investigations [1,9-11]. Especially torque-resisting TADs (PI and BA) revived interest in [3,5-7,22] and the anterior palate as an insertion site, even for MS [8,12]. About several indications has been reported [7,8,12], in case reports [13,14], in reviews [15,16] or experts opinions [17-21]. But yet, an actual frequency has not been clarified and also not which indications should to be placed. Besides in-vitro or animal-experimental studies, previous investigations reported about a defined sample, but not about the relation of patients treated with skeletal anchorage to the whole patient collective. Therefore no conclusion can be drawn how many patients actually seem to require skeletal anchorage or how many treatment cases with skeletal anchorage were relatively performed. We found that in only 4.2% of all patients treated within the investigated period the indication for skeletal anchorage (PI) was placed. So due to missing data, no comparison to other findings can be made. Considering the amount of required treatment, determining the need to propose skeletal anchorage, it was pointed out that most of the patients (71.4%) were treated in the upper dentition, in 48.2% was the right and left upper side (two quadrants) treated. But 28.6% of the sample was also treated in the mandible (by applying Class II elastics to mesialize lateral teeth in the mandible), meaning treatment in three or four quadrants. This is emphasized by looking at the treatment challenges. We found that two third of the found sample offered malocclusions which required multi-functional treatment purposes; or in other terms, the indication for treating patients skeletally anchored was mostly placed when multi-functional treatment purposes were necessary.

Therefore, against the common indication of TADs inserted in the anterior palate to conduct orthodontic treatments solely in the maxilla as shown in previous publications, the range of indications should to be extended.

Regarding the single indications, most frequently sagittal tooth movements were conducted (in 85.7% of the sample). This is similar to the findings of Jung et al. [7]. They reported in their RCT study of the spectrum of indications for PIs in treatment concepts involving immediate and conventional loading. The main conducted tooth movement was also in the sagittal plane [7]. Subdividing the movement, the conventional loading group presented a greater amount of distalization than of mesialization, the immediate loading group showed an equal distribution [7]. Other published data reported solely about distalization of the lateral upper teeth [12,14]. Our investigation showed that in most of the cases mesialization of lateral teeth was performed (44.6%), and secondly distalization (21.4%). Therefore, in most of the cases the indication for treatment with skeletal anchorage was placed when patients required treatment to mesialize their lateral teeth.

Evaluating the amount of re-fixation after loss of bonded patches we found that nearly all incidences occurred when one provider was treating. The failure rate of PI insertions is highly correlated with the surgeon’s experience as described by Jung et al. [3]. Therefore we concluded that those bondings may also be sensitive to the performing provider.

The present study was a retrospective evaluation of the frequency and range of indications, but the treatment outcome was not assessed. Therefore no statement of the efficiency can be made, which leads to further investigations to evaluate this topic.

## Conclusion

4.2 % of all treated patients within the investigated period required orthodontic treatment with skeletal anchorage (PI). We could show that the palatal implant was mainly used for multi-functional anchorage purposes, and in one third of those patients also used as skeletal anchorage for treatment in the mandible. Regarding the indications we found that in most of the cases sagittal tooth movement was conducted, and instead of distalization was most frequently mesialization of lateral teeth performed.

We concluded that the indication for skeletal anchorage should be placed in selected cases and primarily when requiring multi-functional anchorage challenges.
